# Editorial: Methods in treating heart failure—device and surgery approach

**DOI:** 10.3389/fcvm.2024.1426133

**Published:** 2024-05-22

**Authors:** Jamshid H. Karimov, Antonio Loforte

**Affiliations:** ^1^ Department of Biomedical Engineering, Lerner Research Institute, Cleveland Clinic, Cleveland, OH, United States; ^2^ Cleveland Clinic Lerner College of Medicine, Case Western Reserve University, Cleveland, OH, United States; ^3^ Kaufman Center for Heart Failure, Heart, Vascular, and Thoracic Institute, Cleveland Clinic, Cleveland, OH, United States; ^4^ Department of Surgical Sciences, University of Turin, Turin, Italy; ^5^ Transplant Center, City of Health and Science Hospital Turin, Turin, Italy

**Keywords:** surgical techniques, heart failure, mechanical circulatory support, device therapies, surgical innovations

**Editorial on the Research Topic** Methods in treating heart failure—device and surgery approach

Finding ways to deal with/treat patients with heart failure (HF) is one of the most serious issues facing the healthcare industry today. A primary contributor to cardiovascular mortality that affects approximately 64 million people worldwide, the prevalence of HF, is rising rapidly, even in developing nations (1). Despite exponentially evolving improvements and accelerating technological leaps for all healthcare industry stakeholders, treatment of cardiovascular disease has remained woefully stagnant. In healthcare, HF has held primacy as the most overwhelming burden on both patient morbidity and mortality, arguably driven by high incidence and its considerable detrimental effects. The following acknowledgement of works submitted to this topic, which are all critical works on HF, intend to provide a broad range of medical, therapeutic, and device options and to reflect the present landscape of surgical and technological armaneraium.

Nair addressed the need for improved risk prediction models using artificial intelligence (AI) technology for evaluations of the right ventricular failure after left ventricular device (LVAD) implantation, which remains a major cause of morbidity and mortality in this patient population. Nair provided compelling reviews of existing risk prediction scores, which included primary diagnostic features as well as limitations.

Cardoso et al., summarily reviewed new medical device-based (i.e., mechanical circulatory support) therapies, as well as the various mechanisms for decompensated HF. The article's classification scheme, collectively classified as (DRI_2_P_2_S), has been proposed to address the advanced device-based therapies by their mechanism of action: **D**ilators (increase venous capacitance), **R**emovers (direct removal of sodium and water), **I**notropes (increase left ventricular contractility), **I**nterstitials (accelerate removal of lymph), **P**ushers (increase renal arterial pressure), **P**ullers (decrease renal venous pressure), and **S**elective (selective intrarenal drug infusion).

Wang et al., reported on the first in-man application of the Liwen RF™ ablation system. This open label, one-arm, prospective, non-randomized study used the system for treatment of drug-resistant hypertrophic obstructive cardiomyopathy. At follow up, amongst those patients excluded from either surgical myectomy (for now, considered the “gold-standard” treatment for most patients) or alcohol septal ablation, the Liwen RF™ demonstrated a significant reduction in left ventricular outflow obstruction and symptom relief.

Yu et al., described results from *in vivo* studies (*n = 2*) of hemodynamics and potential mechanisms that drive pulmonary circulation in status of ventricular fibrillation. Upon completion of implanting the continuous-flow LVAD (CF-LVAD) in ovine models, the HeartCon Ventricular Assist Device was used. Overall, study-findings underline the importance of the atrial rhythm and function for the circulation maintenance in patients with ventricular fibrillation post-CF-LVAD.

In their original research article, Wang X et al. described their experiences with a minimally invasive, transverse aortic-constriction model (C57BL/6J mice), investigating pressure overload-induced cardiac remodeling and HF. With specifically calibrated instrumentation, this method was found suitable for high-precision aortic contraction in mice that need high reproducibility and low post-operative mortality, holding potentially important research value with regards to cardiac remodeling and HF experimentation.

Funamoto et al. reported the group's early clinical report which shared clinical experience and patient outcomes with the next-gen surgical Impella 5.5. Patients with surgically implanted axillary Impella 5.5 showed optimal short-term survival rates. Axillary placement of the blood pump was used in a multitude of clinical indications (e.g., bridging strategy to durable support, implant LVAD and heart transplant, or perioperative support for high-risk cardiac surgery), all with excellent outcomes.

Zhou et al., investigated the exploratory efficacy of the D-Shant device for interatrial shunting in treating HF with reduced ejection fraction (HFrEF) and HF with preserved ejection fraction (HFpEF). The team of researchers also chose to study the predictive value of biventricular longitudinal strain for functional improvement in such patients. Improvements in both clinical- and functional-status were observed (at 6 months, post-implantation of atrium shunt device).

Schmitto et al. reported on the first clinical use of the left atrial appendage (LAA) for epicardial micrograft transplantation during LVAD implantation (in a 61-year-old male patient with dilated cardiomyopathy). The LAA micrograft transplant was successfully applied epicardially, in conjunction with LVAD implantation. In addition to potential therapeutic benefits, their approach facilitates gathering mechanistic proof of remodeling efficacy at all observed levels (i.e., functional, molecular, and structural).

Lee et al. investigated the differential outcomes of catheter ablation vs. medical treatment in patients with atrial fibrillation (AF) and HF. The cohort study was stratified by different left ventricular ejection fractions (LVEFs), New York Heart Association class ≥II, and different AF-types. Analysis showed improved LVEF, improved 6-min walk distance, less AF recurrence, and lower all-cause mortality. In favor of catheter ablation vs. medical treatment (in AF patients with HF and LVEF of 36%–50%), less HF hospitalization was observed by investigators. This was seen in AF patients with HF and LVEF ≤50% and LVEF ≤35%.

In a retrospective, single-center, observational study, Ma et al. analyzed the feasibility and outcomes of conduction system pacing (CSP) in HF patients with a severely reduced LVEF of less than 30% (HFsrEF). The CSP was found feasible and safe in patients with HFsrEF and was associated with significantly better clinical and echocardiographic outcomes.

With the underlying objective to conduct a dynamic and longitudinal bibliometric analysis of HF, Kuang et al. provided a comprehensive overview of machine learning applications in HF-associated diseases; the perspective of using AI in HF diagnosis and treatment has been emphasized. Dixit and Amsterdam discussed how revascularization should be prioritized at index hospitalization for newly diagnosed HFrEF patients at high risk for coronary artery disease (not presenting with acute coronary syndrome). Ultimately, the authors propose an algorithm for evaluation of ischemic cardiomyopathy in hospitalized patients with newly diagnosed HFrEF, utilizing strategies that could optimize the guidelines and directions of medical therapy outcomes.

Pyka et al., investigated potential benefits of mechanically supported revascularization for heart transplant candidates. The researchers shared a specific case-report, featuring a 53-year-old male heart transplant candidate with type 1 diabetes mellitus. In the study, the subject was initially considered unsuitable for revascularization and had already qualified for heart transplantation. The operating heart team opted for a high-risk mechanically supported percutaneous coronary intervention for revascularization. Several months post-procedure, the patient was no longer listed as a candidate for heart transplant. Overall, their findings suggested that—in select cases—a more thorough assessment of myocardial viability, with potential revascularization strategies, is critical for these patient populations.

Miyagi et al. showed anatomical- and virtual-fittings of two small-sized (i.e., pediatric and infant patients) continuous-flow total artificial heart pumps (CFTAHs) in congenital heart surgery patients. Performed at Cleveland Clinic on pediatric cardiac surgery patients (*n* = 40), the study used 3D-models of pediatric [P-CFTAH] and infant [I-CFTAH] blood pumps. An important landmark in pediatric blood-pump research, the investigators successfully demonstrated optimal dimensions for each of the pumps and proved them to be feasible in all enrolled patients, including those who were under 10 kg at the time of evaluation assessment.

Shen et al. retrospectively analyzed a total of 460 patients to elucidate the prognostic significance of serum albumin and thereby find the creatinine ratio (ACR) in patients receiving heart transplantation for end-stage HF. Their article also discussed efforts to identify potential correlations between ACR and the prognosis of heart transplantation (with optimal cut-off values), purportedly estimating prognosis in this complex patient population.

Overall, the underlying intention of this Frontiers topic was to discuss the HF therapeutics and treatment option landscape. From covering a representative array of sophisticated clinical and engineering tools currently utilized to investigate clinical gaps in treating HF, to discussing the growing number of viable, novel devices, to evaluating the sophisticated surgical approaches, our selected works are representative but by no means conclusive. Further research is needed to determine long-term evolution of any of the devices; for example, the observed shift in patient phenotypes over time would continue to imply that more devices and treatment strategies should be elaborated. Another such area to examine would be these data points in heart transplant candidates who are less ill and not exclusively at end-stage HF. A regular continuation of research—clinical and data analysis—in addition and development is critical in order to evolve and enable the next generation of surgical techniques and device-based therapies. With time, these research efforts should be able to achieve effective neutralization of device-related complications and to drastically improve overall patient outcomes.
